# “YiJinJing, Wohu Pushi” Posture‐Voice Therapy for Dysarthria in Parkinson's Disease Patients Following Subthalamic Nucleus Deep Brain Stimulation: A Randomized Controlled Trial

**DOI:** 10.1111/cns.70652

**Published:** 2025-11-18

**Authors:** Mei Yang, Xin Sun, Jin Yan, Zhitong Zeng, Yuyan Tan, Shiqing Yan, Yong Wang, Linbin Wang, Chuanxin M. Niu, Dianyou Li

**Affiliations:** ^1^ Center for Functional Neurosurgery, Department of Neurosurgery, Ruijin Hospital Shanghai Jiao Tong University School of Medicine Shanghai China; ^2^ The Affiliated Chuzhou Hospital of Anhui Medical University Chuzhou Anhui China; ^3^ Department of Rehabilitation Medicine, Ruijin Hospital Shanghai Jiao Tong University School of Medicine Shanghai China; ^4^ Department of Neurology, Ruijin Hospital Shanghai Jiao Tong University School of Medicine Shanghai China; ^5^ Shanghai Institute of Inheritance and Daoyin Medicine Shanghai China; ^6^ Department of Traditional Chinese Medicine, Ruijin Hospital Shanghai Jiao Tong University School of Medicine Shanghai China; ^7^ Department of Psychiatry University of Cambridge Cambridge UK

**Keywords:** deep brain stimulation, dysarthria, Parkinson's disease, traditional Chinese medicine, YiJinJing, Wohu Pushi

## Abstract

**Background:**

Dysarthria in Parkinson's disease (PD) is difficult to treat, especially post–subthalamic nucleus deep brain stimulation (STN‐DBS), as therapies like LSVT show variable efficacy and limited accessibility. “Yi Jin Jing, Wohu Pushi” posture‐voice therapy (YJJ‐WPVT), emphasizing postural coordination, offers a promising yet underexplored alternative. The objective of this study is to evaluate the effectiveness of YJJ‐WPVT versus Lee Silverman Voice Treatment (LSVT) and no training for dysarthria in PD patients post‐STN‐DBS.

**Methods:**

This is a prospective, parallel‐assignment, unblinded, randomized controlled trial with follow‐up at 6 months. The trial was conducted at the Department of Neurosurgery, Center for Functional Neurosurgery, Ruijin Hospital, Shanghai Jiao Tong University School of Medicine, Shanghai, China. Patients ≥ 18 years with idiopathic PD treated with STN‐DBS, native Chinese speakers, and Voice Handicap Index‐10 (VHI‐10) > 10. Participants were randomized 1:1:1 to YJJ‐WPVT (*n* = 11), LSVT (*n* = 11), or no training (*n* = 12). Both intervention groups received 16 sessions over 4 weeks. LSVT included sustained phonation, pitch glides, and structured speech tasks with daily home practice. YJJ‐WPVT followed a similar 4‐week protocol and emphasized posture correction through lunges, clawing posture, and tiger roar vocalization. Primary outcomes were changes in Voice Handicap Index‐30 (VHI‐30) and sound pressure level (SPL) at 1 month.

**Results:**

Of 68 patients screened, 34 were enrolled (mean age: YJJ‐WPVT 62 [8] years; LSVT 65 [5]; untreated 60 [6]). At 1 month, YJJ‐WPVT showed significantly lower VHI‐30 total scores by five points (95% CI −8.7 to −1.3; *p* = 0.04) versus no training, with a nonsignificant SPL increase (1.8 points; 95% CI −1.3 to 4.9; *p* > 0.05). YJJ‐WPVT improved MPT, jitter, shimmer, and HNR at 1 and 6 months, along with VHI functional subscores. Motor symptoms, swallowing function, and quality of life also improved, with YJJ‐WPVT outperforming LSVT in swallowing and showing slight motor function benefits. Adverse effects (fatigue, hoarseness) were mild and transient.

**Conclusions:**

YJJ‐WPVT is a safe, effective alternative for dysarthria in PD post‐STN‐DBS, with added swallowing and motor benefits. Larger multicenter trials are warranted.

## Graphical abstract

We assessed the effectiveness of “YiJinJing, Wohu Pushi” posture‐voice therapy (YJJ‐WPVT) versus Lee Silverman voice treatment (LSVT) versus no training for dysarthria in patients with Parkinson's disease (PD) after subthalamic nucleus deep brain stimulation (STN‐DBS). The randomized controlled trial showed a significant improvement in the VHI‐30 score after 1 month of YJJ‐WPVT, with no change in SPL. Comparisons with LSVT favored YJJ‐WPVT for swallowing function and showed a slight advantage for motor function.

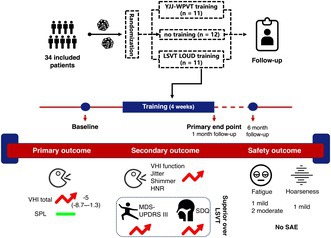

## 1 Background

Parkinson's disease (PD) is a progressive, neurodegenerative disorder marked by key motor and non‐motor symptoms. Among these symptoms, dysarthria affects up to 90% of PD patients as the condition progresses. PD dysarthria, or hypokinetic dysarthria, is characterized by monotone pitch, reduced speech volume and stress, irregular rhythm, imprecise consonants, and a breathy, harsh voice [1]. PD dysarthria significantly impairs daily communication and social interactions, potentially leading to social isolation, mental health issues, and reduced quality of life [2, 3, 4]. Most PD treatments offer limited efficacy for dysarthria; while dopaminergic therapies have variable effects [5, 6, 7], subthalamic deep brain stimulation (STN‐DBS), though established for advanced PD, often worsens dysarthric symptoms [8, 9, 10, 11, 12]. This underscores an urgent need for effective interventions, particularly for individuals with STN‐DBS implants.

Speech therapy is considered effective for managing dysarthria in PD patients. Lee Silverman Voice Training (LSVT) is a widely recognized intensive program designed to enhance vocal loudness in PD patients for effective daily communication [13, 14, 15]. It involves 16 sessions of respiratory and phonatory training over 1 month, delivered by LSVT‐certified speech and language therapists with specialized expertise. The efficacy of LSVT shows promise and is supported by evidence from recent randomized controlled trials (RCTs), demonstrating improvements in speech intelligibility, pitch, loudness, swallowing function, and quality of life [13, 14, 15, 16, 17]. However, LSVT faces limited generalizability across speech outcomes and availability challenges in China, with fewer than 400 certified therapists serving approximately 3 million PD patients. Additionally, STN‐DBS patients exhibited variable responses to LSVT, with no sustained improvements and some experiencing worsening perceptual ratings [18].

Here, we present YiJinJing, a renowned traditional Chinese medicine Daoyin (Chinese physical and breathing exercises, e.g., Eight Trigrams Boxing) [19, 20, 21, 22], increasingly recognized for its therapeutic potential in managing musculoskeletal pain, improving balance, and supporting overall health [23, 24]. The ninth method of YiJinJing, Wohu Pushi (Tiger Hunting Movements), has now been standardized into a structured protocol for PD dysarthria. This method integrates posture coordination and sensorimotor perception during vocalization, utilizing the “crouching tiger” stance as a key element for speech training. Our recent evidence supported the idea that emphasizing posture coordination during speech production yields superior acute effects on respiratory capacity and resonance over LSVT with a single training session, positioning it as a promising alternative for group‐based speech training [25]. However, issues of inadequate intensity, short follow‐up duration, and absence of natural control groups hinder the ability to determine the effects of “YiJinJing, Wohu Pushi” posture‐voice therapy (YJJ‐WPVT).

To this end, we conducted a prospective, single‐center, parallel assignment, unblinded, RCT to assess the clinical effectiveness of YJJ‐WPVT on dysarthria in PD patients following STN‐DBS surgery. The trial primarily assessed the treatment effects of YJJ‐WPVT. An active LSVT group was introduced mid‐trial to allow a head‐to‐head comparison and assess non‐inferiority to the standard of care. Patients were followed up at 1 and 6 months posttreatment to evaluate short‐ and long‐term effects using both objective and subjective outcome measures, reflecting the overall impact on dysarthria and related factors, including swallowing function, mental health, motor severity, and quality of life. The trial was registered at ClinicalTrials.gov (NCT04528147).

## 2 Methods

### 2.1 Study Design and Participants

This is a prospective, single‐center, parallel assignment, unblinded, RCT to assess the effectiveness of YJJ‐WPVT versus LSVT versus untraining for dysarthria in patients with PD after STN‐DBS. Participants were recruited between March 2023 and November 2024 from the outpatient Department of Neurosurgery, Center for Functional Neurosurgery, Ruijin Hospital, Shanghai Jiao Tong University School of Medicine, Shanghai, China.

The inclusion criteria were: (1) patients with idiopathic PD, aged 18 years or older; (2) native Chinese speakers; and (3) a Voice Handicap Index‐10 (VHI‐10) score > 10. The exclusion criteria were: (1) a history of medical conditions affecting swallowing function, such as laryngeal diseases (e.g., vocal nodules, gastroesophageal reflux, or laryngeal cancer); (2) serious psychosis; (3) severe systemic conditions, including cardiac, liver, or kidney diseases, or other critical health issues; (4) dementia (Mini‐Mental State Examination [MMSE] score < 24), inability to comprehend the study protocol, or inability to provide informed consent; (5) respiratory difficulties, fever, lung infections, chronic lung diseases, or abnormal oral structures; and (6) inability to complete the required training tasks for any other reasons.

All participants provided informed consent, and the protocol was approved by the Ethics Committee of Ruijin Hospital, School of Medicine, Shanghai Jiao Tong University (No. 224 of 2022). Training was conducted by certified Yijinjing practitioners (National List of Intangible Cultural Heritage of China, Project No. IX‐2) and licensed LSVT‐LOUD therapists (ONLOUDCH1121‐52, 0822‐18, 0822‐19, 1122‐04).

### 2.2 Randomization and Masking

Participants were randomly assigned in a 1:1:1 ratio to receive YJJ‐WPVT, LSVT, or no training, using permuted block sequences. The sequences were prepared by an independent statistician prior to enrollment and concealed from the recruitment team. Outcomes were assessed by a masked neurologist to avoid bias. Due to the nature of the interventions, masking was not feasible for patients and therapists. All participants maintained their regular dopaminergic treatment throughout the trial, with no adjustments made to DBS parameters. At the conclusion of the trial, untrained PD patients were offered the opportunity to undergo additional training.

### 2.3 Sample Size

Based on the comparable results of a pilot study [26], we estimated a mean (±SD) difference in SPL for the primary outcome of 2.9 ± 2.2. Using this effect size, a sample size of 8.2 patients would be required to achieve 90% power with a significance level of α = 0.05. To account for an anticipated dropout rate of 20%, we planned to enroll 10 patients per group.

### 2.4 Training Oversight

Before training, patients in the YJJ‐WPVT and LSVT groups received detailed explanations of the methods and benefits of speech therapy. All sessions were face‐to‐face guided and supervised by certified speech‐language pathologists (SLPs).

During the 4‐week training period, therapists provided feedback after each session, tracking progress with a standardized recording form. Participants documented daily home practice and submitted weekly logs for review. During home practice, participants continued logging daily practice activities. Weekly WeChat consultations allowed therapists to monitor progress, evaluate training, and ensure goals were achieved.

### 2.5 LSVT

LSVT follows a standardized 4‐week protocol consisting of 16 sessions. The session included 30 min of daily exercises: sustained phonation of the vowel “ah” in a loud, good‐quality voice; pitch glides at high and low frequencies held for 5 s; and reading 10 self‐generated daily phrases with consistent effort and loudness. This was followed by hierarchical speech tasks progressing from single words to conversational speech based on individual goals and interests, and a homework assignment encouraging the use of the practiced louder voice in real‐world communication. Participants performed vocalization tasks while sitting upright on a chair.

### 2.6 YJJ‐WPVT

As a counterpart, YJJ‐WPVT follows a similar 4‐week protocol with 16 sessions to ensure comparable intensity. Training sessions were identical between LSVT and YJJ‐WPVT, including 30 min of daily exercises, hierarchical speech tasks, and a homework assignment. The YJJ‐WPVT protocol emphasized posture correction and speech training through the following components (See Video S1):

1. Lunges: Step forward, bending one knee to 90°–110° with toes aligned forward, keeping the other leg fully extended and torso upright. Alternate legs.
2. Clawing posture: Place hands shoulder‐width apart on a table, fingertips spread and in contact, with elbows straight, shoulders relaxed, and chest lifted.
3. Tiger roar vocalization: Vocalize while extending the neck backward and opening the mouth fully.

### 2.7 Outcomes

The primary outcome was sound pressure level (SPL) and total scores of Voice Handicap Index (VHI)‐30 [27] at 1‐month (primary end point) and 6‐month posttreatment. Previous studies selected either VHI total scores or SPL as primary outcomes: VHI total scores assessed self‐perception of dysarthric severity, while SPL reflected acoustic voice problems [13, 14, 16, 17]. To ensure unbiased results, our study used both as primary outcomes.

Secondary outcome included the VHI subscales, Movement Disorder Society‐Sponsored Revision Unified Parkinson's Disease Rating Scale part III (MDS‐UPDRS III), the GRBAS scale (grade, roughness, breathiness, asthenia, and strain), the 39‐item Parkinson's disease questionnaires (PDQ‐39) [28], the swallowing disturbance questionnaire (SDQ) [29], the Beck depression inventory (BDI)‐2 [30], the Beck anxiety inventory (BAI) [31], and acoustic characteristics of dysarthria. The acoustic measures assessed articulation (SPL), phonation (Jitter, Shimmer, harmonic‐to‐noise ratio [HNR]), prosody (standard deviation of F0 [F0SD]), resonance (diadochokinetic rates [DDK], vowel articulation index [VAI]), respiration (maximum phonation time [MPT]), and overall acoustic quality (dysphonia severity index [DSI]).

All outcome measures were assessed at baseline and followed up at 1 and 6 months.

### 2.8 Adverse Events

Adverse events (AEs) specific to speech therapy are categorized as: (1) Mild: Transient symptoms, alleviated by rest or hydration. (2) Moderate: Persistent symptoms lasting over 48 h, requiring therapy adjustment. (3) Severe: Serious events requiring immediate cessation of therapy, emergency referral, and reporting within 24 h.

Therapists were trained to identify and manage AEs, adjusting therapy as needed. Patients were informed of potential AEs and encouraged to report symptoms. Therapy intensity or frequency was adjusted for those with low tolerance to ensure safety and trial reliability.

### 2.9 Speech Recording and Analysis

All recordings were standardized and conducted by the same experimenter. Speech data were recorded in a quiet room (< 45 dB) with participants seated in a stable chair. Using the lingWAVES system (ATMOS, Germany), recordings were made with a microphone 30 cm from the participant's mouth at 48 kHz and 16‐bit resolution. Participants performed five tasks:

1. Sustain vowels /a/, /i/, /u/.
2. Rapidly repeat syllables /pa/, /ta/, /ka/ six times in one breath.
3. Sustain /a/ at a comfortable volume after a deep breath.
4. Vary /a/ from natural pitch and loudness to maximum and back to minimum.
5. Read two validated Chinese paragraph: *The North Wind and the Sun*.

### 2.10 Statistical Analysis

The primary outcome compared the YJJ‐WPVT group to the untreated group at 1 month. All main analyses adhered to the intention‐to‐treat principle. Baseline‐adjusted changes in VHI total score and SPL at 1 month between groups were analyzed using an ANCOVA model, with posttreatment scores at 1 month as the outcome and baseline scores, age, gender, and motor severity (MDS‐UPDRS‐III total scores) as covariates.

The main secondary comparison involved the YJJ‐WPVT group, LSVT group, and untreated controls, with outcomes assessed at both 1 and 6 months posttreatment. Secondary outcome measurements were adjusted using linear regression models accounting for baseline scores and minimization variables (age, gender, and motor severity). Linear mixed‐effects models were then employed to evaluate group differences and repeated‐measures dependency, incorporating treatment, time, and their interaction as fixed effects, with random intercepts for subjects. Type III analysis of variance (ANOVA) was performed on the fitted model to assess the significance of the fixed effects. Multiple comparisons of secondary outcomes were uncorrected and should be considered exploratory.

We conducted a non‐inferiority test to assess whether the efficacy of YJJ‐WPVT treatment is comparable to that of LSVT treatment. We chose the per‐protocol analysis for the non‐inferiority test to minimize bias toward non‐inferiority. The null hypothesis was that the baseline‐adjusted mean SPL and VHI total scores post‐LSVT treatment would exceed those of YJJ‐WPVT treatment by a non‐inferiority margin. The alternative hypothesis was that the baseline‐adjusted mean SPL and VHI total scores post‐LSVT treatment would fall within the non‐inferiority margin. The non‐inferiority margins were set at −3.5 points for VHI total scores and 1.5 points for SPL, as prespecified based on published trials and the present data. Non‐inferiority was assessed using a one‐sided 5% significance level and two‐sided 90% confidence intervals (95% one‐sided confidence intervals).

Data normality was assessed using the Shapiro–Wilk test. Demographic data were compared across groups using the Kruskal–Wallis *H* test for non‐normally distributed continuous and ordinal variables, the chi‐square test for categorical variables, and the One‐way ANOVA for normally distributed continuous variables.

All statistical analyses and figure plotting were performed using R (version 3.4.3). A *p*‐value of < 0.05 was considered significant.

## 3 Results

### 3.1 Patient Demographics

**FIGURE 1:**
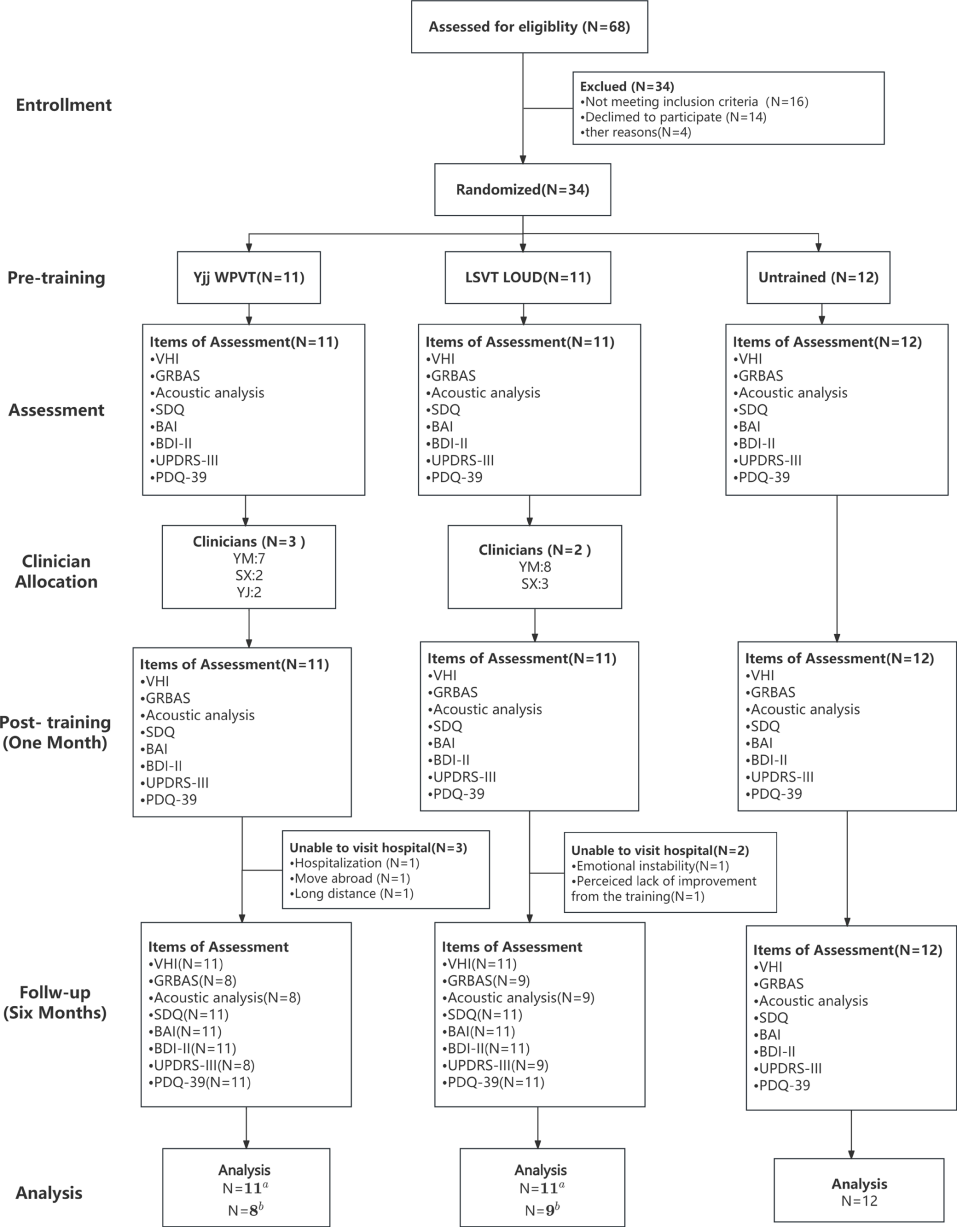
Trial flowchart. ^a^All participants in the “YiJinJing, Wohu Pushi” Posture‐Voice Therapy (YJJ‐WPVT) group and the Lee Silverman Voice Treatment (LSVT) group completed the training. ^b^Completed the 6‐month follow‐up.

Sixty‐eight patients were screened for eligibility, with 34 included and randomly assigned to three groups: YJJ‐WPVT (*n* = 11; mean [SD] age 62 [8] years; 8 [72.7%] male), LSVT (*n* = 11; mean [SD] age 65 [5] years; 6 [54.5%] male), and untreated (*n* = 12; mean [SD] age 60 [6] years; 6 [50%] male) (Figure 1). The groups showed no significant differences in age, gender, disease duration, surgery time, Hoehn‐Yahr stage, or Levodopa Equivalent Daily Dose (LEDD) (Table 1).

**TABLE 1:** Patient demographic information.

Variable	YJJ‐WPVT	LSVT	No treatment
Number	11	11	12
Age, mean (SD), years^a^	62 (8)	65 (7)	60 (5)
Sex, M/F^b^	8/3	6/5	6/6
Years since diagnosis, mean (SD)^c^	11 (3)	12 (4)	13 (4)
Months since surgery, mean (SD)^b^	26 (22)	29 (24)	19 (13)
HY stage, mean (SD)^a^	2.5 (0.7)	2.8 (0.4)	2.3 (1.1)
LEDD, mean (SD)^c^	352 (180)	401 (197)	336 (83)

*Note:* Data are presented as mean ± standard deviation (SD).

Abbreviations: F, female; HY, Hoehn–Yahr scale; LEDD, Levodopa Equivalent Daily Dose; LSVT, Lee Silverman voice treatment LOUD; M, male; YJJ‐WPVT, “YiJinJing, Wohu Pushi” Posture‐Voice Therapy.

^a^ Kruskal–Wallis *H* test was used to analyze non‐normally distributed continuous variables and ordinal variables.

^b^ Chi‐square test was used to analyze categorical variables.

^c^ One‐Way ANOVA was used to analyze normally distributed continuous variables.

### 3.2 Primary Outcomes

At 1 month (primary endpoint), the YJJ‐WPVT group had a significantly lower mean VHI‐30 total score by 5 points (95% CI −8.7 to −1.3, *p* = 0.04) than the untreated group. The mean SPL was 1.8 points (95% CI −1.3 to 4.9, *p* = 0.36) higher but not statistically significant. In contrast, LVST had a significantly lower mean VHI‐30 total score by 9.4 points (95% CI −14.6 to −4.2, *p* = 0.009) and higher SPL by 6.4 points (95% CI 2.2 to 10.6, *p* = 0.02) than the untreated group. No evidence suggested a difference between the YJJ‐WPVT and LSVT groups (VHI‐30: −0.2 points; 95% CI −4.9 to 5.4; *p* = 0.95; SPL: −2.7 points; 95% CI −5.4 to 0, *p* = 0.12), though non‐inferiority of YJJ‐WPVT to LSVT could not be confirmed for either VHI‐30 (*p* = 0.15) or SPL (*p* = 0.96). Linear mixed models over 1‐ and 6‐month repeated measures revealed significant overall effects of YJJ‐WPVT on VHI total scores (−4.1, 95% CI −7.2 to −1.1, *p* = 0.03). At 6 months, no significant differences in primary outcomes were observed among the YJJ‐WPVT, LSVT, or untreated groups (Table 2).

**TABLE 2:** Primary outcome, secondary speech outcomes and additional secondary outcomes.

Outcomes	Mean (SD)			Adjusted mean difference (95% CI), *p* *		
	YJJ‐WPVT	LSVT	No treatment	YJJ‐WPVT vs. no treatment	LSVT vs. no treatment	YJJ‐WPVT vs. LSVT
Primary outcome						
VHI total score (baseline)	44.6 (24.2)	58.5 (25.2)	49.9 (10.1)			
1 month	42.7 (24.0)	55.7 (23.4)	51.4 (12.7)	−5 (−8.7, −1.3), *p* = 0.04	−9.4 (−14.6, −4.2), *p* = 0.009	0.2 (−4.9, 5.4), *p* = 0.95
6 month	44.2 (27.5)	60.1 (27.9)	52.2 (12.2)	−4.4 (−7.9, −0.9), *p* = 0.06	−8.6 (−16.2, −1), *p* = 0.08	−0.4 (−7.9, 7.1), *p* = 0.93
Overall				−4.1 (−7.2, −1.1), *p* = 0.03	−5.1 (−9.7, −0.5), *p* = 0.08	0.2 (−5, 5.4), *p* = 0.96
Secondary outcomes						
VHI functional subscale (baseline)	20.6 (9.3)	24.9 (9.1)	22.2 (3.5)			
1 month	19.2 (7.8)	23.6 (8.5)	22.3 (4.0)	−2.5 (−4.4, −0.6), *p* = 0.04	−3.1 (−5.4, −0.8), *p* = 0.04	−0.4 (−2.5, 1.6), *p* = 0.72
6 month	19.7 (8.9)	26.5 (12.6)	23.1 (3.7)	−3.1 (−4.6, −1.6), *p* = 0.004	−3.1 (−6.6, 0.5), *p* = 0.17	−1.1 (−4.3, 2.1), *p* = 0.59
Overall				−2.1 (−3.5, −0.6), *p* = 0.02	−1.7 (−3.7, 0.4), *p* = 0.19	−0.4 (−2.6, 1.8), *p* = 0.78
VHI physical subscale (baseline)	12.2 (10.9)	15.1 (11.9)	11.5 (5.1)			
1 month	11.9 (11.8)	13.7 (11.4)	11.6 (4.8)	−0.7 (−2.7, 1.3), *p* = 0.58	−2.4 (−4.8, −0.1), *p* = 0.13	1 (−0.7, 2.6), *p* = 0.37
6 month	12.2 (11.8)	14.9 (11.1)	11.8 (4.8)	−0.4 (−2.5, 1.8), *p* = 0.79	−1.8 (−4.9, 1.3), *p* = 0.34	0.7 (−1.9, 3.4), *p* = 0.65
Overall				−0.6 (−2.3, 1.2), *p* = 0.60	−1.3 (−3.2, 0.6), *p* = 0.27	0.8 (−1, 2.7), *p* = 0.46
VHI emotional subscale (baseline)	11.8 (9.7)	18.5 (100)	16.3 (7.4)			
1 month	11.6 (10.5)	18.4 (9.1)	17.6 (8.0)	−1.7 (−3.8, 0.3), *p* = 0.18	−4.3 (−6.6, −2.1), *p* = 0.006	−0.1 (−2.8, 2.6), *p* = 0.94
6 month	11.7 (10.2)	18.6 (10.1)	17.3 (7.7)	−1.4 (−3.6, 0.8), *p* = 0.29	−3.7 (−6.5, −0.9), *p* = 0.04	−0.8 (−3.9, 2.4), *p* = 0.69
Overall				−1.3 (−3, 0.4), *p* = 0.20	−2.3 (−4.1, −0.5), *p* = 0.05	−0.1 (−2.4, 2.2), *p* = 0.95
GRBAS (baseline)	2.8 (2.9)	4 (3.3)	2 (2.0)			
1 month	3 (3.5)	3.2 (3.4)	2.8 (2.7)	−0.9 (−1.6, −0.1), *p* = 0.09	−1.6 (−3.3, 0), *p* = 0.13	1 (−0.4, 2.4), *p* = 0.27
6 month	2.4 (1.9)	3.6 (4.4)	3.8 (3.0)	−1.3 (−3, 0.3), *p* = 0.21	−2.6 (−5, −0.2), *p* = 0.10	0.9 (−1.6, 3.4), *p* = 0.56
Overall				−0.7 (−1.7, 0.2), *p* = 0.21	−0.9 (−2.3, 0.5), *p* = 0.30	0.9 (−0.6, 2.3), *p* = 0.31
SDQ (baseline)	7.0 (3.5)	8.9 (3.8)	5.9 (3.6)			
1 month	5.3 (3.4)	8.1 (3.5)	5.9 (4.3)	−1.8 (−2.9, −0.7), *p* = 0.02	−1.4 (−2.8, −0.1), *p* = 0.09	−1 (−2, 0), *p* = 0.11
6 month	6.8 (3.3)	10.0 (3.5)	6.6 (4.2)	−1 (−2.3, 0.3), *p* = 0.21	0.3 (−1.3, 1.9), *p* = 0.77	−1.5 (−2.7, −0.3), *p* = 0.05
Overall				−1.4 (−2.4, −0.5), *p* = 0.02	−0.8 (−1.8, 0.2), *p* = 0.21	−0.8 (−1.7, 0.1), *p* = 0.14
PDQ‐39 (baseline)	23.9 (9.9)	37.1 (19.0)	24.1 (13.8)			
1 month	23.2 (10.0)	36.1 (19.3)	29.1 (14.9)	−6.2 (−10.4, −2.1), *p* = 0.02	−8 (−12.8, −3.3), *p* = 0.01	0.4 (−1.3, 2.2), *p* = 0.70
6 month	25.7 (10.7)	39.0 (19.7)	31.4 (17.4)	−5.4 (−10.5, −0.3), *p* = 0.10	−8.5 (−14.4, −2.6), *p* = 0.03	0.4 (−1.5, 2.4), *p* = 0.73
Overall				−5 (−8.8, −1.1), *p* = 0.04	−4.4 (−8.3, −0.5), *p* = 0.07	0.3 (−1.2, 1.8), *p* = 0.71
BDI‐2 (baseline)	11.1 (6.7)	15.9 (9.0)	7.4 (11.8)			
1 month	10.8 (6.0)	15.6 (8.0.4)	11.8 (9.4)	−1.7 (−3.8, 0.4), *p* = 0.21	−2.5 (−5.3, 0.2), *p* = 0.15	−0.4 (−1.8, 1.1), *p* = 0.71
6 month	12.0 (6.4)	18.2 (10.1)	13.0 (10.3)	−2.3 (−5.3, 0.7), *p* = 0.23	−0.2 (−4.2, 3.7), *p* = 0.92	−1.3 (−4.2, 1.7), *p* = 0.49
Overall				−1.4 (−3.6, 0.8), *p* = 0.30	−1.3 (−3.5, 0.9), *p* = 0.35	−0.3 (−2.2, 1.6), *p* = 0.81
BAI (baseline)	9.8 (5.3)	12.4 (7.4)	6.6 (7.1)			
1 month	9.9 (4.5)	12.5 (8.2)	7.3 (7.8)	0.2 (−2.6, 2.9), *p* = 0.92	−0.3 (−3.6, 2.9), *p* = 0.88	0 (−1.4, 1.4), *p* = 0.99
6 month	11.4 (5.6)	13.4 (8.8)	7.7 (7.7)	1.4 (−1.6, 4.5), *p* = 0.44	−0.8 (−4.6, 3.1), *p* = 0.75	0.7 (−1.4, 2.8), *p* = 0.59
Overall				0.1 (−2.2, 2.5), *p* = 0.93	−0.2 (−2.5, 2.2), *p* = 0.91	0 (−1.5, 1.6), *p* = 0.99
MDS UPDRS‐III (baseline)	27 (10.0)	29.6 (8.1)	20.5 (7.4)			
1 month	21.3 (9.3)	27.5 (8.9)	25.4 (12.9)	−10.4 (−15.9, −5), *p* = 0.005	−10.1 (−16.9, −3.3), *p* = 0.03	−3.6 (−6.6, −0.7), *p* = 0.06
6 month	22.1 (10.3)	26.7 (9.9)	21.7 (11.7)	−8.4 (−14.1, −2.7), *p* = 0.03	−6.1 (−12.9, 0.6), *p* = 0.15	−3.8 (−9.6, 2), *p* = 0.30
Overall				−8.7 (−13.4, −3.9), *p* = 0.004	−5.5 (−10.4, −0.6), *p* = 0.07	−3.3 (−6.7, 0.1), *p* = 0.13

Abbreviations: BAI, Beck anxiety inventory; BDI, Beck depression inventory; CI, confidence interval; GRBAS, GRBAS scale; LSVT, Lee Silverman voice treatment LOUD; MDS‐UPDRS, Movement Disorder Society‐Sponsored Revision Unified Parkinson's Disease Rating Scale; PDQ‐39, Parkinson's disease‐39 questionnaire; SD, standard deviation; SDQ, swallowing disturbance questionnaire; VHI, voice handicap index; YJJ‐WPVT, “YiJinJing, Wohu Pushi” Posture‐Voice Therapy.

* Analysis adjusted for baseline value (e.g., baseline VHI total) and the minimisation variables (baseline VHI total score, age, gender, and motor severity). Negative difference favors YJJ‐WPVT treatment for comparison of YJJ‐WPVT vs. no treatment and YJJ‐WPVT vs. LSVT; and favors LSVT for comparison of LSVT vs. no treatment. A *p*‐value of < 0.05 was considered significant.

### 3.3 Secondary Speech Outcomes

For VHI subscales, while the YJJ‐WPVT group improved VHI functional subscores at both 1 and 6 months compared to the untreated group, the LSVT group improved VHI functional subscores at 1 month and VHI emotional subscores at both 1 and 6 months compared to the untreated group. Linear mixed models confirmed significant overall effects of YJJ‐WPVT on functional subscores and LSVT on emotional subscores (Table 2).

Acoustic analysis showed significant prolonged MPT at 1 month for both YJJ‐WPVT and LSVT groups, but only for the LSVT group at 6 months. The YJJ‐WPVT group showed significant improvements in jitter, shimmer, and HNR at both 1 and 6 months, whereas the LSVT group showed no significant changes. Particularly, jitter and HNR were significantly better in the YJJ‐WPVT group compared to the LSVT group at 1 month. No significant treatment effects were observed in the other acoustic and speech measurements (Table 3).

**TABLE 3:** Acoustic speech outcomes.

Outcomes	Mean (SD)			Adjusted mean difference (95% CI), *p* *		
	YJJ‐WPVT	LSVT	No treatment	YJJ‐WPVT vs. no treatment	LSVT vs. no treatment	YJJ‐WPVT vs. LSVT
Articulation (primary outcome)						
SPL (baseline)	72.7 (6.2)	71.5 (4.0)	74.2 (3.2)			
1 month	73.0 (4.8)	76.3 (4.1)	71.6 (4.6)	1.8 (−1.3, 4.9), *P* = 0.36	6.4 (2.2, 10.6), *p* = 0.02	−2.7 (−5.4, 0), *p* = 0.12
6 month	70.0 (6.5)	74.8 (6.6)	71.0 (5.2)	−0.7 (−5.4, 3.9), *p* = 0.80	7 (1.2, 12.7), *p* = 0.06	−5.6 (−11.8, 0.5), *p* = 0.16
Overall				1.4 (−1.5, 4.3), *p* = 0.42	3.5 (0.1, 6.9), *p* = 0.11	−2.4 (−5.7, 0.9), *p* = 0.25
Respiration						
MPT (baseline)	7.0 (3.5)	7.9 (4.9)	13.3 (5.8)			
1 month	12.0 (3.9)	10.8 (4.7)	11.4 (4.5)	5.2 (3.3, 7.2), *p* < 0.001	3.6 (1.2, 6), *p* = 0.02	1.5 (−0.4, 3.5), *p* = 0.22
6 month	12.6 (4.8)	10.5 (4.0)	9.7 (3.7)	4.5 (0.7, 8.4), *p* = 0.07	4.1 (1.2, 7.1), *p* = 0.04	2.2 (−1.4, 5.8), *p* = 0.34
Overall				3.2 (1, 5.4), *p* = 0.02	1.9 (0, 3.7), *p* = 0.11	1.4 (−0.8, 3.5), *p* = 0.30
Phonation						
Jitter (baseline)	1.5 (1.3)	0.8 (0.7)	0.6 (0.4)			
1 month	0.4 (0.4)	1.0 (1.1)	0.8 (0.7)	−0.8 (−1.2, −0.4), *P* = 0.006	−0.1 (−0.5, 0.3), *P* = 0.79	−0.9 (−1.4, −0.3), *P* = 0.02
6 month	0.0.4 (0.2)	1.1 (0.9)	1.1 (0.7)	−0.8 (−1.1, −0.4), *p* = 0.005	−0.4 (−0.8, 0), *p* = 0.16	−0.7 (−1.3, −0.1), *p* = 0.07
Overall				−0.5 (−0.8, −0.2), *p* = 0.007	0 (−0.3, 0.2), *p* = 0.83	−0.7 (−1.1, −0.3), *p* = 0.01
Shimmer (baseline)	11.6 (5.5)	7.7 (4.5)	6.9 (1.9)			
1 month	6.1 (3.9)	7.3 (4.6)	7.2 (3.1)	−3.7 (−6.4, −1.1), *P* = 0.03	−1.1 (−4.4, 2.1), *p* = 0.57	−3.5 (−6.5, −0.5), *p* = 0.07
6 month	3.9 (8.6)	10.5 (4.3)	9.6 (3.7)	−1.6 (−4.3, 1.2), *p* = 0.37	−2.6 (−5.4, 0.2), *p* = 0.15	−2.6 (−4.9, −0.4), *p* = 0.08
Overall				−2.5 (−4.4, −0.6), *p* = 0.04	−0.6 (−2.6, 1.4), *p* = 0.62	−2.8 (−4.9, −0.7), *p* = 0.04
HNR (baseline)	10.6 (5.8)	14.5 (6.2)	14.7 (2.9)			
1 month	17.2 (6.8)	14.1 (5.3)	14.1 (3.9)	5.7 (2, 9.4), *p* = 0.02	0 (−3.3, 3.4), *p* > 0.99	6.5 (3, 10.1), *p* = 0.008
6 month	13.8 (5.1)	12.5 (5.4)	10.6 (4.2)	2.9 (0.9, 5), *p* = 0.03	3.6 (0.3, 6.9), *p* = 0.09	2.1 (−0.7, 5), *p* = 0.23
Overall				4.1 (1.7, 6.4), *p* = 0.008	0 (−2.2, 2.2), *p* > 0.99	5.2 (2.8, 8.1), *p* = 0.002
Prosody						
DDK (baseline)	2.9 (1.1)	3.2 (1.0)	4.2 (1.1)			
1 month	3.2 (1.1)	3.6 (1.2)	4.2 (1.4)	0 (−0.8, 0.9), *P* = 0.95	0.8 (−0.2, 1.8), *p* = 0.19	−0.4 (−1, 0.2), *p* = 0.33
6 month	3.8 (1.3)	3.6 (0.7)	3.8 (1.1)	0.5 (−0.7, 1.7), *p* = 0.53	−0.1 (−1.1, 0.9), *p* = 0.86	0.2 (−0.6, 0.9), *p* = 0.74
Overall				0 (−0.6, 0.7), *p* = 0.96	0.4 (−0.2, 1), *p* = 0.29	−0.3 (−0.9, 0.2), *p* = 0.31
F0SD (baseline)	28.8 (9.1)	38.4 (19.9)	44.9 (11.8)			
1 month	29.0 (11.4)	35.0 (11.3)	39.6 (16.0)	4.1 (−6.6, 14.8), *p* = 0.54	−7 (−19.6, 5.5), *p* = 0.37	−1.2 (−7.9, 5.5), *p* = 0.76
6 month	30.3 (12.2)	38.2 (8.9)	35.8 (12.3)	−4.2 (−20.4, 11.9), *p* = 0.67	−0.7 (−12, 10.6), *p* = 0.92	−6.4 (−14.4, 1.6), *p* = 0.21
Overall				2.1 (−5.1, 9.2), *p* = 0.64	−3.3 (−10.6, 4.1), *p* = 0.47	−1.1 (−6.5, 4.4), *p* = 0.75
Resonance						
VAI (baseline)	0.7 (0.1)	0.8 (0.2)	0.8 (0.1)			
1 month	0.9 (0.1)	0.8 (0.2)	0.9 (0.1)	0.1 (0, 0.2), *P* = 0.10	−0.1 (−0.2, 0), *p* = 0.40	0.1 (0.1, 0.2), *p* = 0.02
6 month	0.9 (0.1)	0.8 (0.2)	0.9 (0.1)	0.1 (0, 0.2), *P* = 0.13	0.1 (0, 0.2), *p* = 0.13	0.1 (0, 0.2), *p* = 0.18
Overall				0.1 (0, 0.1), *P* = 0.19	0 (−0.1, 0), *p* = 0.45	0.1 (0, 0.2), *P* = 0.02
Acoustic voice quality						
DSI (baseline)	−4.3 (1.9)	−4.2 (2.0)	−2.3 (1.3)			
1 month	−2.9 (1.9)	−3.8 (2.3)	−2.5 (1.7)	0.7 (−0.8, 2.1), *p* = 0.47	−0.1 (−2.1, 1.8), *p* = 0.90	0.8 (−0.5, 2), *p* = 0.32
6 month	−3.0 (2.1)	−3.9 (1.5)	−3.1 (1.3)	1.5 (0.2, 2.8), *p* = 0.07	0 (−1.3, 1.2), *p* = 0.98	1.1 (0, 2.2), *P* = 0.12
Overall				0.4 (−0.6, 1.4), *P* = 0.47	−0.1 (−1.1, 1), *p* = 0.91	0.7 (−0.3, 1.7), *p* = 0.24

Abbreviations: CI, confidence interval; DDK, diadochokinetic rates; DSI, dysphonia severity index; F0SD, standard deviation of F0; HNR, harmonic‐to‐noise ratio; LSVT, Lee Silverman voice treatment LOUD; MPT, maximum phonation time; SD, standard deviation; SPL, sound pressure level; VAI, vowel articulation index; YJJ‐WPVT, “YiJinJing, Wohu Pushi” Posture‐Voice Therapy.

* Analysis adjusted for baseline value (e.g., baseline SPL) and the minimisation variables (baseline SPL, age, gender, and motor severity). Positive difference favors YJJ‐WPVT treatment for comparison of YJJ‐WPVT vs. no treatment and YJJ‐WPVT vs. LSVT; and favors LSVT for comparison of LSVT vs. no treatment. For jitter and shimmer, however, negative difference favors YJJ‐WPVT treatment for comparison of YJJ‐WPVT vs. no treatment and YJJ‐WPVT vs. LSVT; and favors LSVT for comparison of LSVT vs. no treatment. A *p*‐value of < 0.05 was considered significant.

### 3.4 Additional Secondary Outcomes

Both the YJJ‐WPVT and LSVT groups showed improvements in motor symptoms (MDS‐UPDRS‐III) and quality of life (PDQ‐39) at 1 and 6 months compared to the untreated group, though these effects were attenuated over time. YJJ‐WPVT demonstrated marginally greater improvement in motor severity than LSVT at 1 month. Both YJJ‐WPVT and LSVT showed significant overall effects on PDQ‐39, while only YJJ‐WPVT had a significant overall effect on MDS‐UPDRS‐III scores. The YJJ‐WPVT group had lower SDQ scores at 1 month compared to the untreated group, indicating improved swallowing function. Repeated measures analysis confirmed the overall effects of YJJ‐WPVT on SDQ scores and, more notably, revealed its superiority over LSVT. Neither the YJJ‐WPVT nor LSVT groups showed effects on depression (BDI‐2) or anxiety (BAI) (Table 2).

### 3.5 Adverse Effects

Adverse effects included fatigue (LSVT: 2 [18.2%] mild, 1 [9.1%] moderate; YJJ‐WPVT: 1 [9.1%] mild, 2 [18.2%] moderate), neck tension (LSVT: 3 [27.3%] moderate), hoarseness (LSVT: 3 [27.3%] moderate; YJJ‐WPVT: 1 [9.1%] mild), and mood fluctuations (LSVT: 1 [9.1%] moderate). No serious adverse events were reported.

## 4 Discussion

The trial investigated YJJ‐WPVT's effects on dysarthria in PD patients with STN‐DBS. YJJ‐WPVT improved patient‐reported dysarthria symptoms but did not significantly affect SPL. Its efficacy was comparable to LSVT, though non‐inferiority requires further verification. While benefits persisted for 6 months, therapeutic effects were not significant, suggesting a need for re‐intervention or ongoing exercise. YJJ‐WPVT also improved quality of life, motor function, and swallowing. Compared to LSVT, YJJ‐WPVT showed advantages in swallowing and marginally in motor function. Adverse effects were minor, transient, and manageable. These findings support YJJ‐WPVT as an effective alternative for dysarthria in PD patients with STN‐DBS.

Acoustic analysis revealed distinct effects of YJJ‐WPVT and LSVT on dysarthria, suggesting their potential for tailoring to specific patient groups or training goals. YJJ‐WPVT improves respiration capacity (MPT), phonation control (Jitter, Shimmer, HNR), and primarily enhances the VHI functional domain. In contrast, LSVT increases loudness (SPL), improves respiration capacity (MPT), and mainly benefits the VHI emotional domain. The improvements in respiratory capacity and phonation control may reflect YJJ‐WPVT's focus on posture coordination and sensorimotor control, optimizing respiratory muscle function for increased airflow and precise vocal cord regulation of pitch, volume, and sound quality with improved laryngeal muscle support. Moreover, posture correction during vocalization with YJJ‐WPVT may improve voice production impaired by postural abnormalities commonly observed in PD patients [32].

One of the interesting findings in our study is that YJJ‐WPVT can improve swallowing function and shows an advantage over LSVT. Dysphagia is prevalent in PD and a leading cause of aspiration pneumonia and death, with no established restorative treatment [33, 34, 35, 36]. Pilot studies suggest that LSVT may influence swallowing as a by‐product of voice treatment, potentially impacting the oral and pharyngeal phases of swallowing [16, 17]. While LSVT aims to enhance loudness in daily communication, YJJ‐WPVT focuses on restoring the functionality supporting vocal production through stance posture and axial control. This approach may also improve swallowing by enhancing coordinated muscle control of the aerodigestive tract, respiratory support, respiratory‐swallow coordination, and reducing muscle rigidity and bradykinesia. However, further study focused on swallowing function as the primary outcome is needed to confirm the efficacy of YJJ‐WPVT.

It is noteworthy that our study focused on patients with PD who underwent STN‐DBS, a group characterized by relatively advanced disease and more pronounced axial symptoms. Even with optimal medication and DBS treatment, YJJ‐WPVT further improved motor symptoms. This highlights the importance of rehabilitation in more advanced patients. Untrained patients showed gradually worsening dysarthria and motor symptoms, reflecting disease progression. Although DBS parameters remain unchanged throughout the trial, cumulative deteriorative effects may still occur. Speech outcomes in surgical PD patients are variably affected by STN‐DBS. It depends on factors such as voltage amplitude, frequency, contact location, and potential adverse effects on the cognitive‐motor system [11, 37, 38, 39]. As such, the potential interaction between DBS and YJJ‐WPVT should be considered, as it may amplify the effects of YJJ‐WPVT, although this study did not focus on this aspect. While this study focused on the effects of YJJ‐WPVT on oropharyngeal motor control, YiJinJing, as a balance‐oriented mind–body exercise, is also likely to improve balance and gait in PD. These benefits may be achieved through three mechanisms: enhancement of overall motor function as demonstrated in this study, improvement of dynamic postural control through Tai Chi–like mechanisms [19], and modulation of anxiety and executive function that supports better dual‐task gait performance [21, 24, 40, 41].

Despite the strengths of the study, several methodological limitations should be considered. First, differences in therapy access and intervention formats make blinding unfeasible, introducing potential bias in the expectations and engagement of both participants and specialists. Second, despite efforts to ensure comparable treatment intensity, equal intervention dosage between the active arms is not possible, particularly as YJJ‐WPVT requires more physical engagement, inherently increasing intensity. Therefore, the dose effect cannot be ruled out. Third, results may be influenced by the expertise of the specialists. While LSVT specialists were mostly newly trained, potentially underestimating its efficacy on dysarthria, observed improvements in SPL, VHI, and quality of life align with previous trials and the recent UK‐based multicenter RCT, reinforcing confidence in the findings [13, 15, 26].

## 5 Conclusions

The study indicates that YJJ‐WPVT is an effective and safe alternative therapy for dysarthria in PD patients with STN‐DBS, offering potential additional benefits for swallowing and motor function. A large‐scale, multicenter RCT is warranted to validate its clinical efficacy.

## Author Contributions

Conceptualization: L.W., C.M.N., and D.L. Formal analysis: L.W. and M.Y. Funding acquisition: D.L. and C.M.N. Investigation: M.Y. and J.Y. Methodology: X.S., L.W., C.M.N., and D.L. Project administration: D.L. and C.M.N. Resources: D.L. Supervision: S.Y. and Y.W. Writing – original draft: L.W. and M.Y. Writing – review and editing: L.W., D.L., and C.M.N.

## Ethics Statement

This study was approved by the Ethics Committee of Ruijin Hospital, School of Medicine, Shanghai Jiao Tong University (no. 224 of 2022).

## Conflicts of Interest

The authors declare no conflicts of interest.

## Supporting information


**Video S1:** “YiJinJing, Wohu Pushi” Posture‐Voice Therapy (YJJ‐WPVT) protocol. The training session involves sustained phonation of “ah” in a loud, quality voice, pitch glides at high and low frequencies for 5 s, and posture correction in YJJ‐WPVT treatment.

## Data Availability

The data that support the findings of this study are available on request from the corresponding author. The data are not publicly available due to privacy or ethical restrictions.
